# Supplementary material for: Automated exploitation of the big configuration space of large adsorbates on transition metals reveals chemistry feasibility
Source: Nat Commun. 2022 Apr 26;13:2087. doi: 10.1038/s41467-022-29705-7 (PMC9043206; doi:10.1038/s41467-022-29705-7)
Supplement: Supplementary file 1 — Supplementary Information [file 41467_2022_29705_MOESM1_ESM.pdf]

**Supplementary Information: Automated Exploitation of the Big Configuration Space of Large Adsorbates on Transition Metals Reveals Chemistry Feasibility**

D. Vlachos et al.

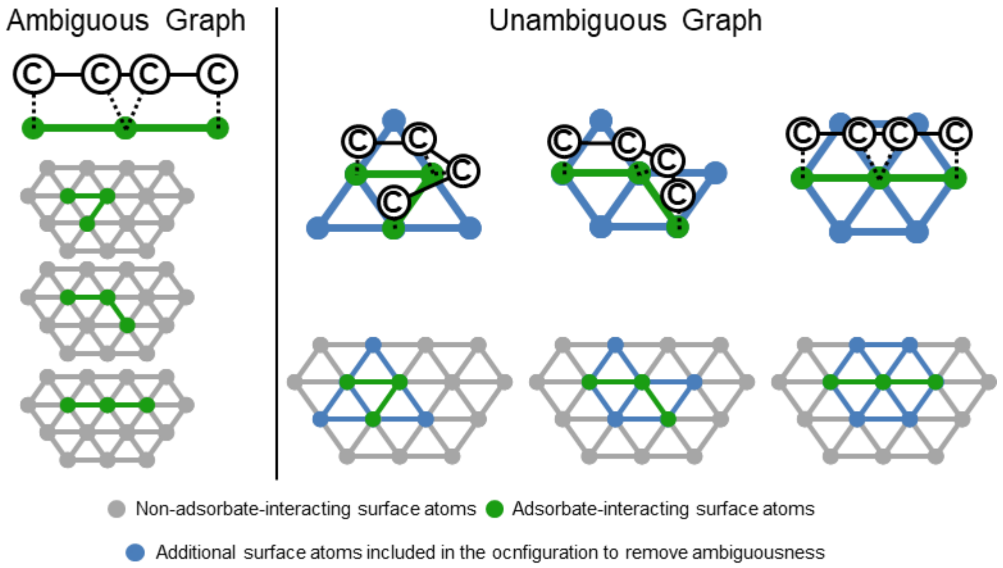


Supplementary Figure 1. Algorithm for trimming the surface atoms while ensuring uniqueness and unambiguousness. The graph transformation is performed on the large lattice. To build a concise graph, surface atoms are removed. A naïve way is to remove all non-adsorbate-interacting surface atoms, but as shown on the left, this results in ambiguous graphs, representing non-isostructural configuration. Instead, surface atoms that are connected to at least 2 occupied surface atoms are retained in the graph, which ensures conciseness as well as uniqueness.

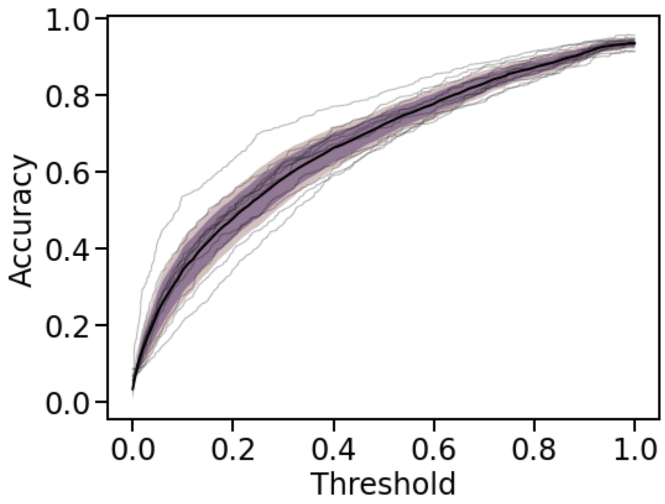


Supplementary Figure 2. Accuracy of the stability prediction model.

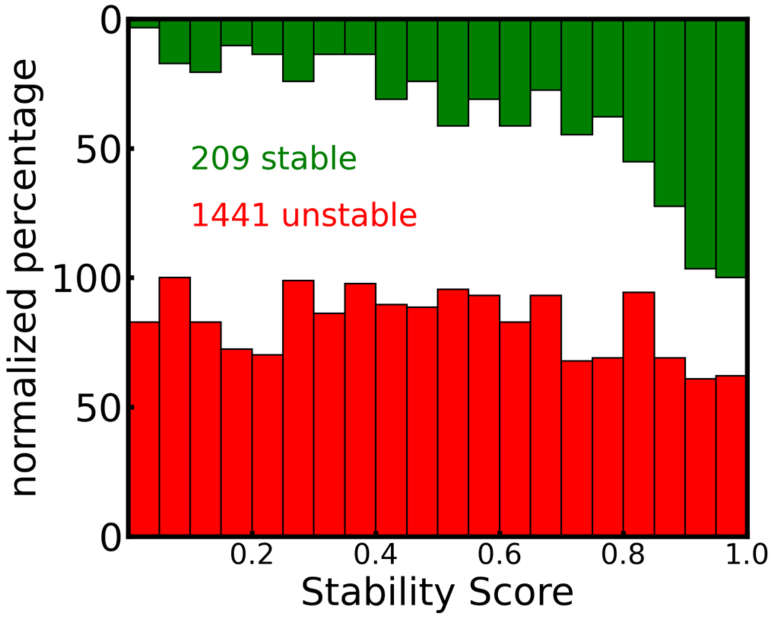


Supplementary Figure 3. The stability prediction scores vs. the density functional theory calculated stability for larger molecules (>3 heteroatoms).50 configurations each with 4, 5, and 6 heteroatoms are sampled for 11 metals.

Supplementary Table 1. Calculated *d*-band center relative to the Fermi level for the metals considered in this study.

| Surface | d-band center |
|---|---|
| Co (0001) | -1.27 |
| Ni (111) | -1.42 |
| Rh (111) | -1.60 |
| Ru (0001) | -1.66 |
| Pd (111) | -1.74 |
| Re (0001) | -1.90 |
| Cu (111) | -2.40 |
| Pt (111) | -2.41 |
| Ir (111) | -2.76 |
| Au (111) | -3.39 |
| Ag (111) | -3.89 |

Supplementary Table 2. Confusion matrix for the stability convergence between high and low fidelity DFT calculations.

| | | High fidelity | |
|---|---|---|---|
| | | Stable | Unstable |
| Low fidelity | Stable | 43 | 20 |
| | Unstable | 9 | 126 |

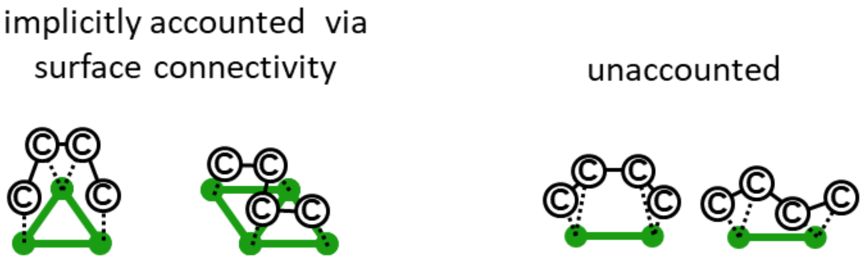


Supplementary Figure 4. Left shows the butadiene examples where cis, trans isomerism is accounted for due to the surface connectivity pattern, whereas the right shows the examples where the isomerism is not accounted for in our current scheme.
